# Birch pollen, air pollution and their interactive effects on airway symptoms and peak expiratory flow in allergic asthma during pollen season – a panel study in Northern and Southern Sweden

**DOI:** 10.1186/s12940-022-00871-x

**Published:** 2022-07-06

**Authors:** Hanne Krage Carlsen, Susanna Lohman Haga, David Olsson, Annelie F Behndig, Lars Modig, Kadri Meister, Bertil Forsberg, Anna-Carin Olin

**Affiliations:** 1 Section of Occupational and Environmental Medicine, School of Public Health and Community Medicine, Institute of Medicine, Sahlgrenska Academy, University of Gothenburg, Medicinaregatan 16A, 40530 Gothenburg, Sweden; 2 Department of Public Health and Clinical Medicine, University Hospital, Sustainable Health, Umeå University, Building 1A, 4st, 901 87 Umeå, Sweden; 3 Section of Medicine, Department of Public Health and Clinical Medicine, University Hospital, Umeå University, Building 1A, 4st, 901 87 Umeå, Sweden; 4 Department of Statistics, USBE, Social Sciences Building Level 2 (ground floor), Umeå University, 90187 Umeå, Sweden

**Keywords:** Birch, *Betula*, PM_2.5_, O_3_, Panel study, Allergic asthma, Pollen season

## Abstract

**Background:**

Evidence of the role of interactions between air pollution and pollen exposure in subjects with allergic asthma is limited and need further exploration to promote adequate preventive measures. The objective of this study was to assess effects of exposure to ambient air pollution and birch pollen on exacerbation of respiratory symptoms in subjects with asthma and allergy to birch.

**Methods:**

Thirty-seven subjects from two Swedish cities (Gothenburg and Umeå) with large variation in exposure to both birch-pollen and air pollutants, participated in the study. All subjects had confirmed allergy to birch and self-reported physician-diagnosed asthma. The subjects recorded respiratory symptoms such as rhinitis or eye irritation, dry cough, dyspnoea, the use of any asthma or allergy medication and peak respiratory flow (PEF), daily for five consecutive weeks during two separate pollen seasons and a control season without pollen. Nitrogen oxides (NO_x_), ozone (O_3_), particulate matter (PM_2.5_), birch pollen counts, and meteorological data were obtained from an urban background monitoring stations in the study city centres. The data were analysed using linear mixed effects models.

**Results:**

During pollen seasons all symptoms and medication use were higher, and PEF was reduced in the subjects. In regression analysis, exposure to pollen at lags 0 to 2 days, and lags 0 to 6 days was associated with increased ORs of symptoms and decreased RRs for PEF. Pollen and air pollution interacted in some cases; during low pollen exposure, there were no associations between air pollution and symptoms, but during high pollen exposure, O_3_ concentrations were associated with increased OR of rhinitis or eye irritation, and PM_2.5_ concentrations were associated with increased ORs of rhinitis or eye irritation, dyspnea and increased use of allergy medication.

**Conclusions:**

Pollen and air pollutants interacted to increase the effect of air pollution on respiratory symptoms in allergic asthma. Implementing the results from this study, advisories for individuals with allergic asthma could be improved, minimizing the morbidities associated with the condition.

**Supplementary Information:**

The online version contains supplementary material available at 10.1186/s12940-022-00871-x.

## Background

Exposure to air pollution can cause a variety of adverse health effects such as respiratory illness, cardiovascular disease and lung cancer, and it has been estimated that approximately 9 million people die each year from causes directly attributed to air pollution [1]. The prevalence of asthma is reported to be 4.3% globally, and as high as 10% in Sweden [2]. The prevalence has been increasing in the last decade [3] and allergic asthma is the most commonly reported form of asthma in Sweden with an estimated prevalence of 7.3% [4].

Exposure to air pollutants such as ozone, particulate matter (PM), and nitric oxides (NO_x_) is associated with increased rhinitis severity [5] and short term exposure can induce airway inflammation, due to increased oxidative stress, and further result in airway hyperresponsiveness in asthmatic individuals and sensitization [6, 7] and poor asthma control [8]. Birch (*betula*) pollen is a common allergen in allergic asthma [9]. Inhalation of pollen grains (who are themselves too large to reach the small airways) results in the rupture of the grain and release of the allergenic content (Bet v1 protein) which activates an immune response, [10] and cause bronchoconstriction in individuals with allergic asthma [11]. Increased allergenicity of birch pollen following exposure to ozone [12] and PM [13] has been reported and it has also been speculated that pollution attaches to the allergen [14]. Anthropogenic air pollution affects the abundance and bioavailability of pollen, and future climate change may affect the duration and severity of pollen seasons [15–18]. Pollen and air pollution peaks often co-occur [19], therefore it is of interest to study pollen and air pollution together. Although synergistic effects or interactions between air pollution and pollen on respiratory health outcomes in individuals with allergic asthma have been reported [14, 20], many uncertainties remain, the estimated risks have a large range, and more studies on this topic are warranted [21, 22].

The aim of the present study was to study associations and interactions between pollen and air pollutants associated with worsening of daily self-reported respiratory symptoms and peak expiratory flow (PEF). Furthermore, we wanted to assess if there are lagged effects of exposure to pollen and air pollution on symptoms reports, study effects in individuals with more severe disease, as indicated by either steroid medication use, or have poorer asthma control.

## Methods

### Study population

Forty-one non-smoking subjects (27 to 73 years of age) with self-reported physician diagnosed asthma and a confirmed birch allergy (positive skin-prick test, phadiatop ≥ 0.35 kU/L) were recruited to participate. 22 subjects were recruited in Gothenburg (Lat. 57° N) in the southern part of Sweden and 17 from Umeå (Lat. 63° N), in northern Sweden. Eventually, 22 and 15 had diary data and were included in the analysis. Subjects with cardiovascular disease or any chronic inflammatory disease were excluded from participation. All participating subjects signed an informed consent agreement, and the study was approved by the Regional Ethical Review Board at the University of Gothenburg (Dnr: 682 − 14).

### Study design

The participating subjects filled out a health diary which included questions about daily symptoms and medication who had been used in previous studies [23, 24] for five consecutive weeks (35 days) during two separate pollen-seasons (2015 and 2016) and once outside the pollen-season (November-December 2015).

The symptoms registered in the diary (Supplementary document D1) were cold, fever, rhinitis, dyspnea and dry cough (yes or no), and allergy medication (yes or no), bronchodilation medicines (compared to their regular dose)” (no, less, normal or more), and asthma symptoms (no, less, normal or more). Peak respiratory flow rate (PEF) measurements were made twice daily throughout the five weeks, in the morning before any medication and before bedtime, with three registered measurements for each occasion.

The means for morning and evening PEF for each subject during the study waves were first calculated (PEF_mo_ and PEF_ev_). Individual deviations of daily performance from each subject’s mean PEF_mo_ and PEF_ev_ per season were then calculated and averaged across the participants to obtain daily mean deviations (∆) for PEF_mo_ and PEF_ev_ values, ∆PEF_mo_ and ∆PEF_ev_.

To avoid error or bias due to effects of respiratory disease epidemics, we excluded days where participants reported having a “fever”. In total, 8 individuals reported “fever” on at least one occasion (46 observations with “fever”, 3133 observations with “no fever” reported). The presence of cold was reported on at least one day by 22 of the participants, with a total of 297 observations of cold, 2855 observations with no cold), but as the symptoms of cold are somewhat similar to those of asthma and allergy, they were retained in the data, but not analysed.

All subjects underwent a clinical examination once within each season and completed an Asthma Control Questionnaire (ACQ) with six questions regarding symptoms and use of bronchodilators and inhaled steroids during the week before the clinical examination and pre-bronchodilator forced expiratory volume in one second (FEV_1_) in categories [25]. The participants also reported their medication usage in the previous month in a questionnaire.

### Exposure

Birch pollen counts were obtained from centrally located pollen monitoring stations using standard methods [26]. Ambient concentrations of the air pollutants NO_x_, O_3_ and PM_2.5_, and meteorological data of relative humidity and temperature were measured at centrally located fixed monitoring stations in the two study centers and obtained from the environmental authorities as previously described [27]. In this study, we used PM_2.5_ as this was available in both locations for the study period. In brief, routinely collected air pollution, pollen, and meteorology data were provided to us from the relevant authorities (Gothenburg: Gothenburg municipality https://goteborg.se/wps/portal/start/miljo/miljolaget-i-goteborg/luft/luftkvaliteten-i-goteborg, and the Pollen Laboratory at Gothenburg University https://www.gu.se/biologi-miljovetenskap/pollen-och-allergier), Umeå Municipality (https://www.umea.se/byggaboochmiljo/boendemiljobullerochluftkvalitet/luftenutomhus/luftkvaliteteniumea.4.250f9659174ae4b97941ae7.html). Relevant lags were calculated and used in the analyses. All participating subject lived within, or in direct vicinity to the city centers in question.

The data were converted to 24-hour means and were assigned to each study date and lagged values were calculated [27]. The birch pollen season was defined as the first occurrence of 5 consecutive days where 4 days had pollen counts > 0 [26] (not dissimilar to the recently proposed pollen exposure threshold from recent position paper) [28].

### Statistical analyses

Descriptive statistics for diary-reported outcome and exposure variables are presented as mean and standard deviations (SD) and categorical variable frequencies were compared with Chi2-tests between control season and pollen seasons. The proportion reporting symptoms, medication use, and mean PEF was reported for each season.

The outcomes were analyzed with mixed models, where subjects were included as a random effect, and climate and air pollution variables were included as fixed effects.

The mean of lag-intervals lag 0–2, and secondarily lag 0–6 were chosen based on previous literature. However, the lag-association between outcomes and pollen and air pollution exposure was also investigated using distributed lag non-linear models (DLNM) [29] at lags 0 to lag 10 with different options for the shape of the lag-associations and levels of adjustments. The models with best (lowest) AIC were selected and plotted (Supplementary Fig. S2) and showed that in most cases, the significant association between exposure and outcome did, in fact, occur at lag 0–2.

In the analysis of morning PEF values, same day pollen exposure was not included as they were deemed of less interest, only exposure lags from the previous day and backwards in time were included (lag1, lag 1–2 and lag 1–5).

Logistic regression was used for analysis of the daily symptoms in asthma-diary and linear regression for PEF. In the main analysis, data from all study seasons were analyzed separately for each study center (reported in the Supplement), then the results were pooled using random effects meta-analysis.

Single-exposure models included the covariates pollen, temperature and relative humidity, dual-exposure models included pairwise combinations of pollen and air pollutants O_3_, NO_x_, and PM_2.5_, temperature, and relative humidity. Multi-exposure models included pollen, all three pollutants, temperature, and relative humidity.

Interaction analysis for pollen and air pollution were first investigated in mixed models with id and study center as random effects with an indicator variable for the presence of pollen season (pollen season vs. control season) for the association between outcomes and air pollutants in all data. Then, using only pollen season data, we investigated interaction between low and high levels of pollen at lag 0–2 (using 100 pollen/m^3^ as a cutoff) and air pollution values.

The sample size power calculation was based analyses of effects in respiratory biomarkers in analyses comparing effects in pollen season and control season. In the current study, each individual had up to 105 measurements (days with diary data), the statistical power is sufficient. In order to estimate effect of disease severity on the response from pollen exposure, stratified sensitivity analyses were performed in individuals who reported using inhalation corticosteroids, nasal steroids, individuals with poor asthma control as measured by ACQ (poor asthma control being defined as a score of ACQ above 1.5) [25]. To determine if drop-out rates affected the results, we also analyzed the individuals who participated in all three waves separately. Data were also analyzed stratified by sex.

The models for PEF were adjusted for autocorrelation by incorporating a first-order autoregressive component. This autocorrelation function is not defined for logistic regression models.

All results are reported per 100-unit pollen and one interquartile range (IQR) change in air pollution concentrations. The results for diary reported symptoms and medication usage are reported as odds ratios (ORs), which express the relative change in the odds associated with change in exposure with 95% confidence intervals (CI). The coefficients for ∆PEF are reported with 95% CI, which express the increase in outcome per increment change in exposure. The interaction terms are reported with their confidence intervals and p-values, and the predicted values of the outcomes at low and high values of the variable pollen at lag 0–2 were plotted. Analyses were performed using the packages “lme4”, “meta”, and “ggeffects” [30–32] in R.

## Results

### Descriptive statistics

Initially, 41 individuals with allergic asthma were recruited for the study, and 37 individuals had information on all key variables of interest and were included in the analysis. The proportion of females was 51%, the mean age was 48 (SD 15) years, and the mean BMI was 26 (SD 5) kg/m2. The participants’ mean ACQ score at baseline was 1.3 (SD 1.1). The regular use of short-acting bronchodilator during the last year were reported by 65% of the participants, long-acting bronchodilator medication by 11% and inhalation corticosteroid use was reported by 68% of the participants (Table 1).

**Table 1:** Demographic characteristics of the participants at baseline

Variables	Gothenburg	Umeå	Total
Subjects, N	22	15	37
Females, n (%)	12 (55%)	7 (47%)	19 (51%)
Males, n (%)	10 (45%)	8 (53%)	18 (49%)
Age, yr (mean ± SD)	44 ± 10	47 ± 13	48 ± 15
Height, cm (mean ± SD)	175 ± 9	172 ± 9	171 ± 8
Weight, kg (mean ± SD)	79 ± 18	78 ± 16	77 ± 15
Body mass index, kg/m2 (mean ± SD)	26 ± 4	26 ± 6	26 ± 5
Birch pollen allergy, n (%)	22 (100%)	15 (100%)	37 (100%)
Asthma Control Questionnaire (mean ± SD)	1.3 ± 1.1	1.3 ± 1.2	1.3 ± 1.1
**Self-reported medication** ^**a**^			
Short-acting bronchodilator, n (%)	18 (82%)	6 (40%)	24 (65%)
Long-acting medication, n (%)	2 (9%)	2 (13%)	4 (11%)
Inhalation steroids medication, n (%)	19 (86%)	6 (40%)	25 (68%)
Nasal steroid, n (%)	7 (32%)	2 (13%)	9 (24%)
Steroid medication (Inhalation and combination steroids), n (%)	19 (86%)	8 (53%)	27 (73%)
Allergy medicine, n (%)	22 (100%)	11 (73%)	33 (89%)

Asthma control questionnaire score calculated as a mean of six questions about asthma symptoms in the last week

^a^Regular usage in the last month or year

Air pollutants and pollen concentrations from stationary measuring stations varied between seasons and study cites. NO_x_ concentrations were lower in Umeå than in Gothenburg during spring in both 2015 and 2016, mean 9.2 µg/m^3^ and 14.6 µg/m^3^ vs. 25.6 µg/m^3^ and 28.7 µg/m^3^ respectively, but higher during control season by 39.5 µg/m^3^ vs. 28.9 µg/m^3^ although the standard deviation for the control season in Umeå was high. O_3_ concentrations were quite higher in Umeå than in Gothenburg during spring 2016, 86.8 µg/m^3^ vs. 53.9 µg/m^3^. PM_2.5_ concentrations were two to three times as high in Gothenburg as in Umeå in all seasons (Table 2, Fig. S1).

**Fig. 1:**
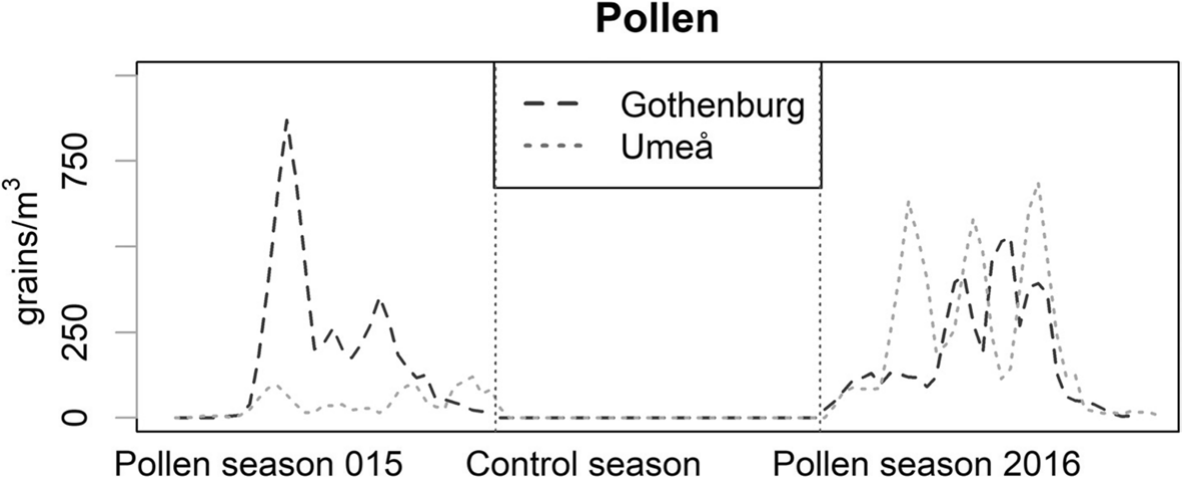
Pollen concentrations in the two study centres (3-day moving average) during the study period

Pollen concentrations were higher in Gothenburg than in Umeå spring 2015 (maximum pollen value 1087 vs. 210), but during spring 2016 pollen concentrations were marginally higher in Umeå than Gothenburg (maximum pollen value 964 vs. 950) (Table 2; Fig. 1). Temperatures and relative humidity also differed between locations and seasons reflecting the geographic differences between the locations (Table 2).

**Table 2:** Daily averages of exposure in the two study centers during the study waves (mean ± standard deviation)

	Wave	Gothenburg	Umeå
**NO**^**x**^ **(µg/m**^**3**^**)**	1 (Pollen season)	25.6 ± 14.7	9.2 ± 5.0
	2 (Control season)	28.9 ± 18.3	37.5 ± 30.7
	3 (Pollen season)	28.7 ± 19.0	14.6 ± 6.6
**O**^**3**^ **(µg/m**^**3**^**)**	1 (Pollen season)	66.0 ± 12.3	63.5 ± 7.7
	2 (Control season)	52.0 ± 15.7	40.0 ± 19.4
	3 (Pollen season)	53.9 ± 13.8	86.8 ± 29.0
**PM**_**2.5**_ **(µg/m**^**3**^**)**	1 (Pollen season)	9.0 ± 6.3	3.7 ± 2.7*
	2 (Control season)	6.9 ± 4.3	2.1 ± 1.3
	3 (Pollen season)	7.1 ± 3.9	4.6 ± 2.6
**Pollen (grains per m**^**3**^)	1 (Pollen season)	180 ± 254	43 ± 49
	2 (Control season)	-	-
	3 (Pollen season)	176 ± 221	231 ± 270
**Temperature (°C)**	1 (Pollen season)	9.1 ± 1.9	1.0 ± 4.2
	2 (Control season)	7.5 ± 3.5	10.0 ± 3.6
	3 (Pollen season)	10.8 ± 4.4	5.9 ± 5.0
**Relative humidity (%)**	1 (Pollen season)	72.5 ± 11.0	75.1 ± 11.2
	2 (Control season)	85.5 ± 6.9	95.1 ± 6.8
	3 (Pollen season)	64.7 ± 12.9	68.9 ± 12.6

Data are measured at centrally located ambient measuring stations.

After initially testing individual lags from 0 to 3 (data not shown), lag 0–2, and lag 0–6, for both pollen and air pollution, and afterwards investigating lag-associations with DLNM methods, it was found that the highest ORs for dry cough and allergy medication were at lag 0–2 and lag 0–6, and these lags are reported onwards. For PEF_mo_, we report results for the mean of the previous day and the day before that, lag 1 to lag 2 instead of lag 0 to lag 2. After excluding observations where participants reported having a fever, there were 3112 observations. In the diaries, the most reported symptoms were rhinitis or eye irritation, reported on 27.5% of days during the study period, followed by dry cough, reported on 26.4% of all days in the study. Participants reported taking allergy medication on 40.7% of the days. Using more bronchodilation medicines than normal was reported on 9.2% of days (Table 3). Mean morning PEF_mo_ was 436 mL (SD 88) and mean PEF_ev_ was 444 (SD 92).

**Fig. 2:**
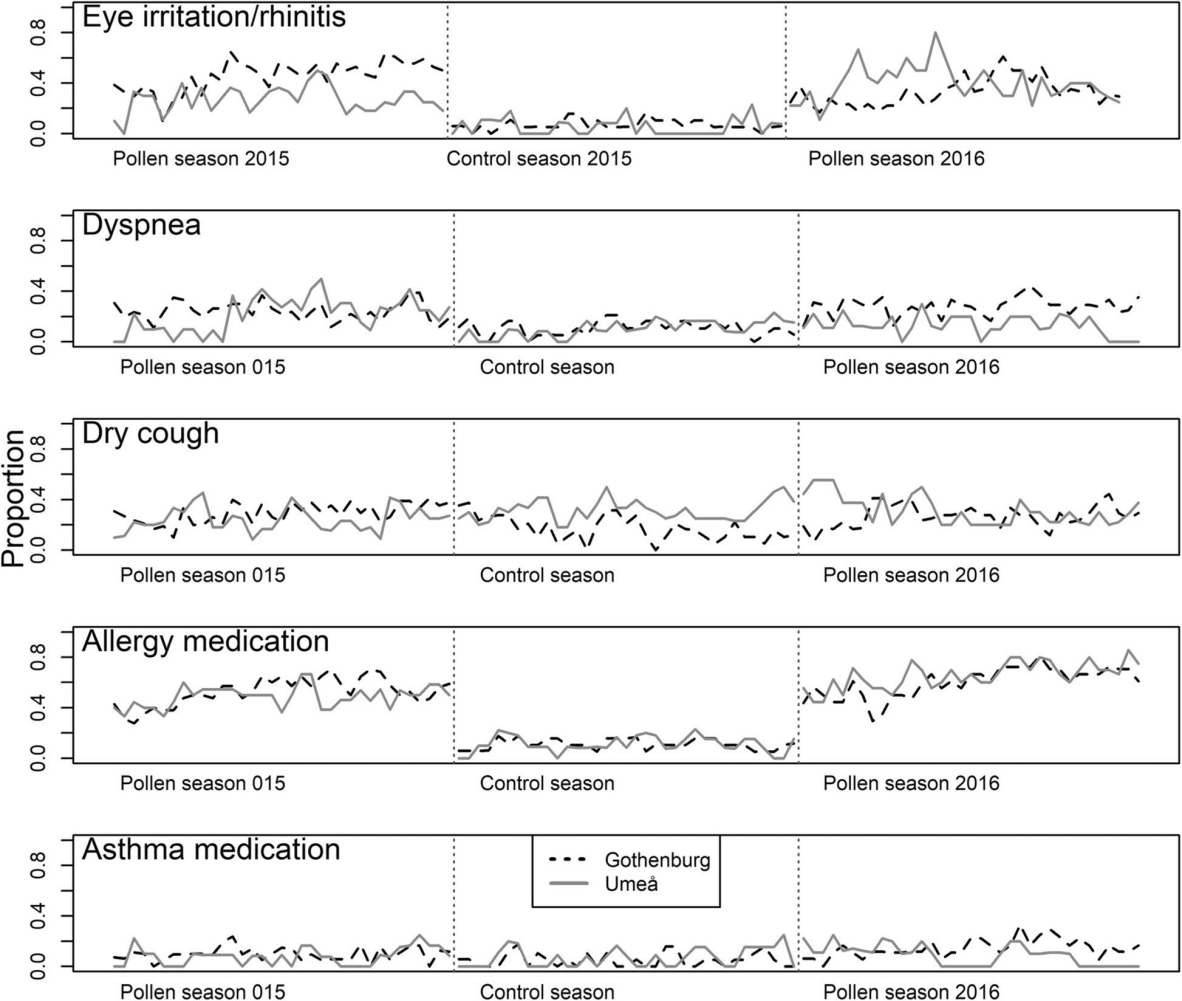
Proportion of diary-reported respiratory symptoms and medication use during the study period. For eye irritation/rhinitis, dyspnea, dry cough, allergy medication, proportion reporting “Yes” vs. “No”. For asthma medication, reporting “more than yesterday” vs. “No”, “Less”, or “Same”

Rates of symptoms and medication usage were higher during pollen season compared to the control season (visualized in Fig. 2). PEF-values during pollen season were not statistically significant different from control season, with *p*-value = 0.15 for PEF_mo_ and *p*-value = 0.24 for PEF_ev_ (data not shown).

**Table 3:** Prevalence of daily diary-reported symptoms, medication use, and mean (SD) of peak expiratory flow in the study population (*n* = 37) during the three study waves

	Wave 1 (1068 observations)	Wave 2 (1066 observations)	Wave 3 (978 observations)	Total (3112 observations)
**Symptoms** ^**a**^	**n (%)**	**n (%)**	**n (%)**	**n (%)**
Eye irritation/rhinitis	649 (60.9%)	68 (6.4%)	368 (37.7%)	851 (27.5%)
Dyspnea	252 (23.8%)	112 (10.6%)	215 (22.9%)	579 (19.0%)
Dry cough	296 (27.9%)	239 (22.7%)	272 (28.9%)	807 (26.4%)
**Allergy medication** ^**a**^	547 (51.3%)	118 (11.2%)	581 (61.9%)	1246 (40.7%)
**Bronchodilating medication**				
No	389 (36.5%)	474 (44.9%)	355 (37.6%)	1218 (39.7%)
Less	46 (4.3%)	64 (6.1%)	14 (1.5%)	124 (4.0%)
Normal	533 (50.0%)	455 (43.1%)	454 (48.1%)	1442 (47.0%)
More	98 (9.2%)	62 (5.9%)	121 (12.8%)	281 (9.2%)
**Peak expiratory flow (PEF)**	**Mean (SD)**	**Mean (SD)**	**Mean (SD)**	**Mean (SD)**
PEF_mo_ (mL)	434 (81)	441 (90)	434 (93)	436 (88)
PEF_ev_ (mL)	442 (85)	448 (93)	442 (97)	444 (92)

*PEF*_*mo* _ Peak expiratory flow measured in the morning,  *PEF*_*ev* _ Peak expiratory flow measured in the evening

^a^For eye irritation/rhinitis, dyspnea, dry cough, allergy medication, “Yes” vs. “No”. For bronchodilating medication, reporting “more than yesterday” vs. “No”, “Less”, or “Same”

### Effects on symptoms and medication

In the pooled analysis (Table 4), pollen exposure at lag 0–2 was statistically significantly associated with increased ORs of reporting rhinitis or eye irritation OR 1.44 (95% CI 1.36–1.52) per 100 pollen/m^3^ pollen in the single-exposure model, and lower, but still statistically significant ORs in dual-exposure and multi-exposure models (City-specific ORs are shown in Table S2a). The same was true for pollen exposure at lag 0–6 which was associated with increased ORs of rhinitis or eye irritation in single-exposure models by OR 1.67 (95% CI 1.57–1.78). PM_2.5_ was significantly associated with increased rhinitis and or irritation in multi-exposure models by OR 1.16 (95% CI 1.02; 1.32).

**Table 4:** OR of diary-reported symptoms and medication use associated with exposure to pollen at lag 0–2 and lag 0–6 and air pollution at lag 0–2 exposure to pollen and air pollutants in single-, pairwise-, and multi-exposure models with 95% confidence interval (pooled results). Significant results are indicated with bold font

Model adjustment	Pollen – Lag 0–2	Pollen – Lag 0–6	NO_x_ – Lag 0–2	O_3_ – Lag 0–2	PM_2.5_ – Lag 0–2
	**OR (95% CI)**	**OR (95% CI)**	**OR (95% CI)**	**OR (95% CI)**	**OR (95% CI)**
**Rhinitis or eye irritation**					
Adjusted for Pollen	**1.44 (1.36; 1.52)**	**1.67 (1.57; 1.78)**			
+ NO_x_	**1.17 (1.02; 1.35)**	**1.28 (1.08; 1.53)**	1.06 (0.86; 1.29)		
+ O_3_	**1.14 (1.02; 1.26)**	**1.23 (1.12; 1.35)**		0.81 (0.56; 1.17)	
+ PM_2.5_	**1.18 (1.01; 1.37)**	**1.35 (1.06; 1.72)**			0.88 (0.78; 1.01)
+ NO_x_, O_3_, PM_2.5_	**1.25 (1.16; 1.35)**	**1.39 (1.27; 1.51)**	0.71 (0.42; 1.19)	1.02 (0.34; 3.04)	**1.16 (1.02; 1.32)**
**Dyspnea**					
Adjusted for Pollen	**1.17 (1.10; 1.25)**	**1.23 (1.09; 1.37)**			
+ NO_x_	1.04 (0.96; 1.14)	**1.12 (1.02; 1.23)**	0.75 (0.54; 1.05)		
+ O_3_	1.03 (0.95; 1.12)	**1.09 (1.00; 1.20)**		1.06 (0.69; 1.61)	
+ PM_2.5_	1.01 (0.93; 1.10)	1.08 (0.98; 1.19)			0.91 (0.55; 1.50)
+ NO_x_, O_3_, PM_2.5_	1.03 (0.95; 1.13)	1.09 (0.99, 1.20)	0.79 (0.54; 1.15)	0.96 (0.66; 1.40)	1.03 (0.81; 1.33)
**Dry cough**					
Adjusted for Pollen	1.05 (0.90; 1.24)	1.08 (0.85; 1.35)			
+ NO_x_	**1.10 (1.02; 1.18)**	**1.14 (1.05; 1.24)**	0.97 (0.72; 1.31)		
+ O_3_	**1.09 (1.01; 1.17)**	**1.14 (1.05; 1.24)**		**1.15 (1.00; 1.31)**	
+ PM_2.5_	1.07 (0.99; 1.16)	**1.14 (1.05; 1.24)**			**1.18 (1.05; 1.33)**
+ NO_x_, O_3_, PM_2.5_	**1.09 (1.01; 1.17)**	**1.15 (1.05; 1.28)**	0.99 (0.72; 1.35)	**1.14 (1.09; 1.19)**	1.01 (0.97; 1.06)
**Allergy medication**					
Adjusted for Pollen	**1.58 (1.44; 1.74)**	**1.85 (1.60; 2.14)**			
+ NO_x_	**1.23 (1.12 1.35)**	**1.16 (1.08; 1.24)**	**0.72 (0.52; 1.00)**		
+ O_3_	**1.19 (1.08; 1.30)**	**1.15 (1.07; 1.23)**		**1.29 (1.02; 1.62)**	
+ PM_2.5_	**1.15 (1.01; 1.26)**	**1.16 (1.07; 1.23)**			**1.15 (1.02; 1.31)**
+ NO_x_, O_3_, PM_2.5_	**1.17 (1.07; 1.29)**	**1.15 (1.06; 1.25)**	**0.74 (0.59; 0.92)**	1.01 (0.78; 1.32)	**1.25 (1.07; 1.46)**
**Bronchodilating medication**					
Adjusted for Pollen	**1.15 (1.08; 1.21)**	**1.23 (1.16; 1.32)**			
+ NO_x_	1.07 (0.87; 1.32)	1.10 (0.85; 1.42)	0.84 (0.81; 0.88)		
+ O_3_	1.06 (0.87; 1.30)	1.09 (0.83; 1.43)		1.02 (0.77; 1.34)	
+ PM_2.5_	1.07 (0.97; 1.30)	1.10 (0.82; 1.49)			0.97 (0.97; 0.99)
+ NO_x_, O_3_, PM_2.5_	1.07 (0.86; 1.33)	1.12 (0.84; 1.50)	0.81 (0.62; 1.07)	1.04 (0.72; 1.49)	1.09 (0.86; 1.37)

For eye irritation/rhinitis, dyspnea, dry cough, allergy medication, “Yes” vs. “No”. For bronchodilating medication, reporting “more than yesterday” vs. “No”, “Less”, or “Same”

Results from pooled with meta-analysis of mixed model results for pollen (single-exposure), pollen and pairwise combinations of pollen and either NO_x_, O_3_ and PM_2.5_, and multi-exposure models adjusted for pollen. All models were adjusted for relative humidity and temperature, with identification number as a random effect. Associations for are reported per 100 grains/m^3^ for pollen and per city-specific IQR for pollutants (Göteborg: NO_x_ 20.0 µg/ m^3^, O_3_ 22.9 µg/ m^3^, PM_2.5_ 3.3 µg/ m^3^, Umeå: NO_x_ 14.7 µg/m^3^, O_3_ 24.1 µg/ m^3^, PM_2.5_ 2.8 µg m^3^)

Dyspnea was only significantly associated with pollen exposure in single-exposure models by OR 1.17 (95% CI 1.10–1.25) for exposure at lag 0–2 and OR 1.23 (95% CI 1.09–1.37) for exposure at lag 0–6. There were no statistically significant associations between air pollutants and dyspnea.

Dry cough was associated with pollen exposure at lag 0–2 in dual-exposure models with NO_x_ by OR 1.10 (95% CI 1.02–1.18) and with O3 by OR 1.09 (95% CI 1.01; 1.17) and in multi-exposure models by OR 1.09 (95% CI 1.01–1.17). For pollen exposure at lag 0–6 there were statistically significant associations in dual-exposure models adjusted for O_3_, PM_2.5_ by 1.14 (95% CI 1.05–1.24) and in multi-exposure models by OR 1.15 (95% CI 1.05–1.28). Exposure to O_3_ at lag 0–2 was statistically significantly associated with dry cough by OR 1.15 (95% CI 1.00-1.31) per IQR O_3_ in models with pollen, and by 1.14 (95% CI 1.09–1.19) in multi-exposure models. Dry cough rates were associated with PM_2.5_ in pairwise models by OR 1.18 (95% CI 1.05–1.25) per IQR PM_2.5_ (Table 4).

Exposure to pollen at lag 0–2 was associated with increased use of allergy medication in single-exposure models by OR 1.58 (95%CI 1.44–1.74). In dual- and multi-pollutant models, the ORs were reduced but remained significant. For lag 0–6 pollen exposure there was a significant increase in allergy medication reports in single-exposure models by OR 1.85 (95%CI 1.60–2.14) and a similar reduction in the dual-and multi-exposure models was noted. Exposure to NO_x_ at lag 0–2 was associated with significantly reduced ORs of allergy medication usage in both dual-and multi-exposure models, with OR at 0.72 (95%CI 0.52-1.00) and OR 0.74 (95%CI 0.59–0.92) respectively. O_3_ exposure was associated with increased use of allergy medication with OR 1.29 (95% CI 1.02; 1.62) in dual-exposure models. PM_2.5_ was associated with increased allergy medication usage in dual-exposure model by OR 1.15 (95% CI 1.02–1.31) and in the multi-exposure model by OR 1.25 (95% CI 1.07; 1.46) (Table 4).

Pollen exposure was statistically significantly associated with increased use of bronchodilating medication only in single-exposure models by OR 1.15 (95% CI 1.08–1.21) at lag 0–2 and lag 0–6 by OR 1.23 (95% CI 1.16–1.32). There were no associations between use of bronchodilating medication and pollen in dual- or multi pollution models, nor with NO_x_, O_3_ and PM_2.5_ air pollution (Table 4).

### Effects on PEF

In the pooled analysis, there were no statistically significant associations between pollen at lag 0–2, lag 0–6 and ΔPEFmo at any adjustment level, but all estimates were negative.

ΔPEF_ev_ and pollen at lag 0–2 were statistically significantly associated in single-exposure models by -1.24 (95% CI -2.45- -0.04) and in dual-exposure models adjusted for PM_2.5_ by -0.93 (95% CI -1.70-0.17) and by -0.93 (95% CI -1.71-0.15) mL per 100 pollen/m^3^ in the multi-exposure model. For ΔPEFev, there were no statistically significant associations with air pollution (Table 5, city-specific estimates are shown in Table S2b).

**Table 5:** Associations (Β) between peak expiratory flow (PEF) in the morning and evening (PEF_mo_ and PEF_ev_) and exposure to pollen (per 100 grains) and air pollutants in single-, pairwise-, and multi-exposure models with 95% confidence interval (pooled results). Significant results are indicated with bold font

	Pollen lag 0–2	Pollen lag 0–6	NO_x_	O_3_	PM2.5
	**β (95% CI)**	**95% CI**	**95% CI**	**95% CI**	**95% CI**
**ΔPEF**_**mo**_ **(mL)**					
Adjusted for Pollen	-1.10 (-2.86; 0.66)	-1.00 (-3.00; 1.01)	-	-	-
+ NO_x_	-1.06 (-3.04; 0.92)	-0.97 (-3.20; 1.27)	-0.16 (-3.21; 2.88)	-	-
+ O_3_	-1.07 (-2.90; 0.76)	-0.97 (-3.05; 1.11)	-	1.54 (-0.92; 4.00)	-
+ PM_2.5_	-1.10 (-2.67; 0.47)	-1.20 (-3.09; 0.70)	-	-	**2.16 (0.23; 4.09)**
+ NO_x_, O_3_, PM_2.5_	**-1.05 (-2.78;-0.68)**	-1.17 (-3.19; 0.86)	-0.43 (-2.48; 1.63)	0.18 (-1.70; 2.06)	**2.14 (1.01; 3.26)**
**ΔPEFev (mL)**					
Adjusted for Pollen	**-1.24 (-2.45; -0.04)**	-1.24 (-3.88; 1.40)	-	-	-
+ NO_x_	-1.25 (-2.53; 0.03)	-1.26 (-4.01; 1.48)	0.40 (-0.97; 1.77)	-	-
+ O_3_	-1.22 (-2.48; 0.04)	-1.24 (-3.88; 1.40)	-	0.66 (-1.24; 2.55)	-
+ PM_2.5_	**-0.93 (-1.70; -0.17)**	-0.72 (-2.30; 0.86)	-	-	2.88 (-1.80; 7.56)
+ NO_x_, O_3_, PM_2.5_	**-0.93 (-1.71; -0.15)**	-0.73 (-2.25; 0.80)	0.17 (-1.65; 1.31)	-0.27 (-2.35; 1.81)	3.21 (-1.27; 7.68)

ΔPEF: deviation from each individual’s mean of each season. Exposure at lag 1–2 for PEF_mo_ and lag 0–2 for PEF_ev_

Results from pooled with meta-analysis of mixed model results for pollen (single-exposure), pollen and pairwise combinations of pollen and either NO_x_, O_3_ and PM_2.5_, and multi-exposure models adjusted for pollen. All models were adjusted for relative humidity and temperature, with identification number as a random effect. Associations are reported per 100 grains/m^3^ for pollen and per city-specific IQR for pollutants (Göteborg: NO_x_ 20.0 µg/ m^3^, O_3_ 22.9 µg/ m^3^, PM_2.5_ 3.3 µg/ m^3^, Umeå: NO_x_ 14.7 µg/m^3^, O_3_ 24.1 µg/ m^3^, PM_2.5_ 2.8 µg m^3^)

### Interactions between pollen and air pollutants

**Fig. 3:**
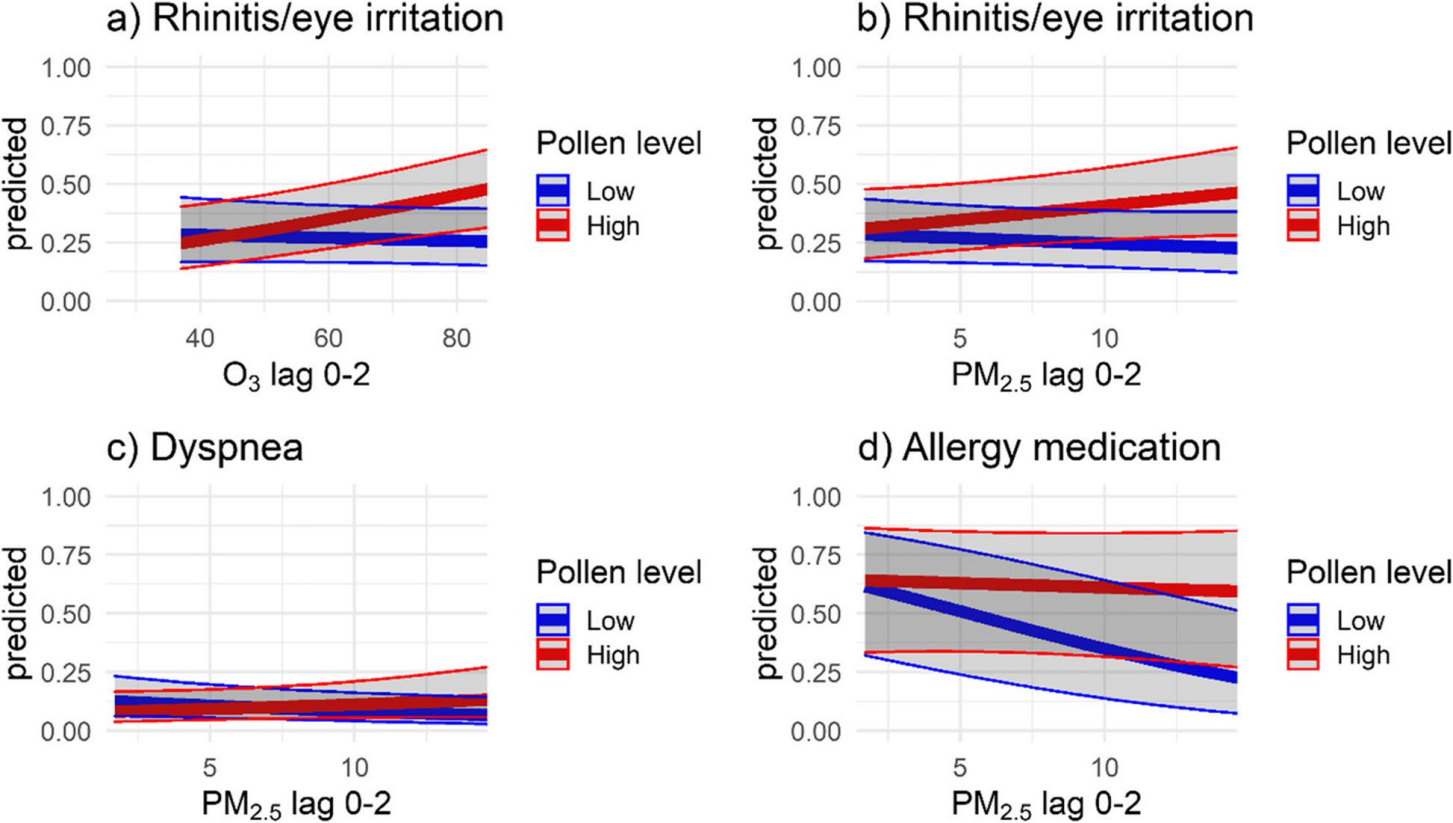
Predicted marginal effects (proportions) of significant interactions between birch pollen levels (below or above 100 grains /m^3^) and pollutants (per µg/m3) on symptoms and allergy medication usage. Footnote: For eye irritation/rhinitis, dyspnea and allergy medication, “Yes” vs. “No”. X-axis from 5^th^ to 95^th^ percentile. Results are from mixed models with identification number and city as a random effect. In addition to pairwise combinations of air pollution and pollen concentration indicator at lag 0–2, the models are adjusted for temperature and relative humidity. Results are reported per global pollutant IQR (NO_x_ 16.4 µg/m^3^, O_3_ 15.2 µg/m^3^, PM_2.5_ 4.7 µg/m^3^)

We observed no interactions between pollen season and air pollution on symptoms, medication usage or PEF when applying a crude measure of pollen season (yes or no), but when categorizing pollen at lag 0–2 into low and high pollen concentrations, there were statistically significant interactions with air pollution for symptoms and medication usage (all interaction term coefficients and p-values are found in Table S3). There were interactions for rhinitis or eye irritation between pollen concentrations and O_3_ by OR 1.45 (95% CI 1.14–1.84) per IQR and with PM_2.5_ by 1.41 (95% CI 1.04–1.92) per IQR. Also, for dyspnea and allergy medication pollen levels interacted with PM_2.5_ concentrations, by ORs 1.56 (95% CI 1.10–2.21) and 1.72 (95% CI 1.13–2.64) per IQR increase in pollutant. The predicted ORs at low and high pollen levels are illustrated in Fig. 3a-d.

PM exposure was negatively associated with decreased allergy medication in the low-pollen exposure scenario, and we speculate.

Additionally, there were indications of pollen-air pollutant interactions (with p-values below 0.1 but above 0.05) for dyspnea, bronchodilating medication and O_3_ (Table S3, Fig. S3).

The autocorrelation adjustment performed for PEF did not change effect estimates of lag 0–2 exposure.

### Sensitivity analysis

In the sensitivity analyses, in observations from individual with poor asthma control (ACQ > 1.5), the estimated association between exposure and rhinitis or eye irritation was higher than in the total data (OR 1.29 (95% CI 1.12–1.48) vs. OR 1.22 (95% CI 1.14–1.31). For other symptoms and PEF, the estimates were similar or lower than in total data (Table S4) and for allergy and asthma medication, the estimates were lower, although the confidence intervals overlapped with the estimates from the total data. In individuals who used inhaled corticosteroid medication (ICS users, *n* = 25), lower ORs were seen for all symptoms compared to the total population, although reports of rhinitis or eye irritation were still statistically significantly associated with pollen exposure (Table S4). In ICS users, there were no associations with ΔPEFev or ΔPEFmo. In individuals who used nasal steroids (NS users, *n* = 9), the ORs for rhinitis or eye irritation and allergy medication in association with pollen counts were higher than in any other group. The results obtained from individuals who participated at all three waves were similar to the total data (Table S4). Stratifying by sex, pollen exposure was only significantly associated with dyspnea, asthma medication usage in female subjects, but the only significant interaction between exposure and sex was observed for bronchodilating medication, where the effects were stronger in females. Morning and evening values of ΔPEF were reduced in both males and females, but the reduction in ΔPEF_mo_ did not reach statistical significance in males (Fig. S4).

## Discussion

In this asthma-diary panel study with data from two different pollen-seasons, we found that symptom prevalence was increased during birch pollen season, and that pollen exposure at lag 0–2 and lag 0–6 was associated strongly with prevalence of most symptoms of allergic asthma as well as medication usage (Table 3). Regarding air pollutants, O_3_ was associated with dry cough and increased use of allergy medication in dual-exposure models. PM2.5 was associated with rhinitis or eye irritation and with allergy medication in multi-exposure models and with dry cough in dual-exposure models. Evening-measured values of PEF were associated with pollen, and PM_2.5_ was associated with morning-measured PEF in multi-exposure models (Table 5). An increase in NOx was in most analyses unexpectedly associated with lower risks. This phenomenon can sometimes be explained by the negative correlation between NO_x_ and O_3_, and the reduction in risk disappears after adjusting for O_3_ .In this case the adjustment did not remove the tendency, and one explanation could be that variations in NO_x_ levels are more local around the central monitoring station than the fluctuations in ozone exposure.

Our study was designed to investigate if pollen exposure increased susceptibility to air pollution, however, we acknowledge that the causal mechanism could be opposite; that air pollution increased the susceptibility to pollen. However, with the current study design, we are unable to test this, as we have no scenario where there is no air pollution.

We investigated effects at different lags with DLNM methodology and found that lag 0–2 or lag 0–6 was relevant in most cases, but that in some cases, for example for PEF_mo_, there could be an additional effect at lag 4–5 which could be investigated in future studies (Fig. S2).

The associations between pollen exposure and lower, upper respiratory symptoms, and rhinitis or eye irritation symptoms in our study are consistent with those from a recent review and meta-analysis of pollen exposure on symptoms in individuals with allergy or asthma. The authors report that 10 pollen/m3 increase in exposure was associated with increased lower respiratory symptoms by 1%, upper respiratory or ocular symptoms by 6.6%, and any symptom of allergy or asthma increased 2%. PEF was not associated with exposure [33]. In a study of mobile-phone app reported symptoms, birch pollen exposure was associated with 3% increase in respiratory symptoms and 4% increase in eye/nose symptoms per 10 pollen/m3 [34].

In addition to previous reports of associations between short term air pollution exposure and severe outcomes such as increased emergency room visits and hospital admissions for asthma [21], or symptom severity recorded during MD visits [35], previous studies report increased asthma symptoms in association with air pollution: PM_2.5_ exposure was associated with increase in mobile-phone app reported respiratory symptoms by 6% per 10 µg/m^3^ [34]. An association between symptoms of allergy and short-term air pollution exposure during pollen season were indicated in a panel studies of individuals with allergy, but the results did not reach statistical significance [36]. After exposure in a road tunnel, asthmatic subjects had increased response to an allergen provocation test with significant correlations between increased asthma symptoms and NO_2_, but not PM_2.5_ exposure [37]. In a controlled study of exposure to diesel exhaust and allergen, the effect of exposure to diesel exhaust was augmented with simultaneous allergen exposure [20].

In our study, we observed only moderate sex-differences in the relationship between pollen and symptoms, where pollen exposure was associated with increased risk of asthma medication use in females, but not in males (Fig. S4). A similar observation was made in a study of asthma symptoms and personal sampler-measured exposure [38] where asthma symptoms during daytime was associated with exposure to oxidants (NO_2_ and O_3_) in women, but not in men, suggesting that females may be more susceptible to effects of air pollution than males.

For PEF, a statistically significant increase of 2.16 (95% CI 0.23–4.09) mL per IQR (3 µg/m^3^) PM_2.5_ in morning PEF was observed in our study, where a recent review found that in asthmatic individuals, PEF was reduced 0.56 L/m per 10 µg/m^3^ PM_2.5_ [39] and a recent review found reduction of 2% per 10 pollen/m^3^, but the time of day (morning or evening) was not taken into account [33]. The unexpected direction of association for PM_2.5_ and PEF could be due to coinciding pollen and PM_2.5_ peaks [19] where pollen exposure compels individuals with asthma to use more bronchodilating medication which reduces airway restriction (Table S4).

In our study, statistically significant pollen – air pollution interactions (interaction *p* < 0.05), where air pollution effects where significantly higher during moderate to high levels of pollen compared to low or no pollen, were present for rhinitis or eye irritation and PM_2.5_ and O_3_, asthma symptoms and NO_x_. In a recent study of self-reported symptoms via a mobile phone app, Bédard and colleagues (2020) observed significant associations between rhinitis symptoms and O_3_ during grass pollen season, but not birch pollen season. PM_2.5_ was statistically significantly associated with rhinitis during the pollen season of one year, but not the other year in the study [40]. In another study of mobile phone app-recorded symptoms, O_3_ concentration led to an increased symptom severity during the birch pollen season, but the association was not significant after adjusting for climate [41]. In a panel study with 15 individuals with allergy, there was an increase in symptom severity associated with exposure to air pollution during pollen season, but the estimates did not reach statistical significance [36].

In a recent review of pollen or fungal spore-air pollution interactions, it was concluded that although interactions had been shown or indicated in time series studies, most of the existing did not consider groups at risk, and there were no studies of adults with allergic asthma [22] making our study unique in that context.

The generalizability of our results to the previous literature on pollen-air pollution interactions in asthma is limited as much of it pertains to more severe outcomes such as asthma emergency room visits or hospital admissions. Also, the few available panel studies apply heterogenous methodologies [21, 22]. Some previous research on pollen-air pollution interaction have compared associations with air pollution during and outside pollen season [40, 41], which did not yield significant results in our study (data not shown), where we entered short-term pollen categories in the interaction models. We speculate that the lag 0–2 pollen exposure is a more precise measure, and that exposure during the whole pollen season was too variable (especially during wave 1 in Umeå, see Fig. 1) to obtain significant results.

In our study, the main results are presented as pooled results (with random effect meta-analyses) from separate analyses of each study center, as we knew of differences in air pollution levels, temperature, as well as recruitment procedures in the two study locations. In the interaction analysis, we entered city as a random effect in the model. For PEF, we tested for autocorrelation by entering an autoregressive term, but for the reported lag 0–2 results, this procedure did not alter the results. There is no definition of this term for logistic regression, so this could not be tested in the analysis of symptom scores.

Our study population was well-defined at baseline, with allergy confirmed with skin-prick tests, but asthma was not confirmed with formal testing as in some other studies [36]. There are indications that some of the cases in our study population had mild disease, e.g., not using any asthma medication which could reduce the strength of our results (Table 1). The results from the sensitivity analysis which was stratified by medication status underlines that our sample had sizable heterogeneity with respect to disease severity. This fact has some consequences for the observed effect size. The sample size deduced based on power calculation of respiratory biomarkers, but as the power in this analysis was increased by the many measures of each individual, the study is unlikely to be underpowered.

In our study, exposure to both pollen and air pollution was measured at central urban background stations, which induces some uncertainty as exposure levels obtained at fixed monitoring stations does not accurately reflect each subject’s personal exposure level, but are rather used as a proxy for exposure in this study. However, this study has a time series element, and so, the day-to-day fluctuations in exposure, which can be expected to be similar across a city, are similar at the monitoring station and the study participants locations. Furthermore, studies have shown central monitoring stations to be a reasonable proxy for personal exposure to birch pollen [42]. Also, monitoring station data correlated well with personal exposure in the study [27]. The panel data collected both in, and outside, pollen season ensured a substantial exposure contrast. The start of the pollen season measured in the study were determined by the first 5-day period with 4 days with pollen counts over 0 [26]. Newer definitions are available which could improve the exposure assessment [28]. However, using this definition may have caused us to choose a slightly premature date for the pollen season in 2015, which was also very mild (Fig. 1; Table 1). We speculate that pollen counts during that season were too low to have an effect, but no universally accepted thresholds of effect have been reported [33].

We observed negative, or protective associations between NO_x_ exposure and symptoms. However, the often-negative correlation between NO_x_ and O_3_ could skew the analysis. However, we calculated a combination value for oxides, O_x_, and used that in the analysis, which did not change the results for pollen. The coefficients associated with O_x_ was statistically significant for bronchodilating medication and morning PEF and in both cases indicated a positive or protective effect of O_x_.

## Conclusions

Our results show that a substantial proportion of allergic asthma symptoms can be attributed to pollen exposure. Also, in addition to direct effects of pollen on symptoms in allergic asthma, pollen exposure increases susceptibility to adverse respiratory effects of exposure to PM_2.5_ and O_3_.

Implementing the results into advisories could improve their predictive power, which could minimize the morbidities associated with allergic asthma and allergy. Considering the number of affected individuals, this could be of substantial benefit to public health. Also, reduction of pollution levels should be priority to improve health in this susceptible population.

## Supplementary Information

**Additional file 1.**

## Data Availability

The data are available for other researchers upon reasonable request and pending ethics approval.

## Abbreviations

ACQ: Asthma Control Questionnaire
NO_x_: Nitrogen dioxides
O_3_: Ozone
PEF: Peak Expiratory Flow
PM: Particle matter
