# Supplementary material for: Birch pollen, air pollution and their interactive effects on airway symptoms and peak expiratory flow in allergic asthma during pollen season – a panel study in Northern and Southern Sweden
Source: Environ Health. 2022 Jul 6;21:63. doi: 10.1186/s12940-022-00871-x (PMC9258213; doi:10.1186/s12940-022-00871-x)
Supplement: Supplementary file 1 — Additional file 1. [file 12940_2022_871_MOESM1_ESM.docx]

# Supplementary material

## Table S1 Correlation matrix for exposure variables (Pearson) with p-values

|  | Pollen (grains/m^3^) | NO_x_ (μg/m^3^) | O_3_ (μg/m^3^) | PM_2.5_ (μg/m^3^) | Temperature (° C) | | Relative humidity (%) |
| --- | --- | --- | --- | --- | --- | --- | --- |
| **GBG (n=97)** | *r* (*p*) | *r* (*p*) | *r* (*p*) | *r* (*p*) | *r* (*p*) | | *r* (*p*) |
| Pollen (grains/m^3^) | 1 |  |  |  |  | |  |
| NO_x_ (μg/m^3^) | 0.14 (0.180) | 1 |  |  |  | |  |
| O_3_ (μg/m^3^) | 0.06 (0.530) | -0.53 (<0.001) | 1 |  |  | |  |
| PM_2.5_ (μg/m^3^) | 0.02 (0.575) | -0.11 (0.304) | 0.36 (<0.001) | 1 |  | |  |
| Temperature (° C) | 0.36 (<0.001) | -0.17 (0.090) | 0.46 (<0.001) | 0.40 (<0.001) | 1 | |  |
| Relative humidity (%) | -0.50 (<0.001) | -0.14 (0.161) | -0.14 (0.185) | 0.08 (0.410) | -0.49 (<0.001) | | 1 |
|  |  |  |  |  |  | |  |
| **Umeå (n=103)** | Pollen (grains/m^3^) | NO_x_ (μg/m^3^) | O_3_ (μg/m^3^) | PM_2.5_ (μg/m^3^) | Temperature (° C) | Relative humidity (%) | |
| Pollen (grains/m^3^) | 1 |  |  |  |  | |  |
| NO_x_ (μg/m^3^) | -0.16 (0. 11) | 1 |  |  |  | |  |
| O_3_ (μg/m^3^) | 0.29 (0.003) | -0.54 (<0.001) | 1 |  |  | |  |
| PM_2.5_ (μg/m^3^) | 0.11 (0.281) | -0.03 (0.738) | 0.30 (0.002) | 1 |  | |  |
| Temperature (° C) | 0.32 (0.011) | -0.60 (<0.001) | 0.62 (<0.001) | 0.37 (<0.001) | 1 | |  |
| Relative humidity (%) | -0.34 (<0.001) | 0.45 (<0.001) | -0.68 (<0.001) | -0.18 (0.065) | -0.63 (<0.001) | | 1 |
| Correlation between 24-hour means of exposure variables. | | | | | | | |

## Table S2a City specific estimates: OR of diary-reported symptoms and medication use associated with pollen exposure at lag 0-2. Adjusted for pollutants in pairwise and multipollutant models (per 100 grains/m^3^). Significant results are indicated with bold font.

| Model | **Gothenburg**  **OR 95% CI** | | |  | **Umeå**  **OR 95% CI** | | |
| --- | --- | --- | --- | --- | --- | --- | --- |
| **Rhinitis or eye irritation** | |  |  |  |  |  |  |
| Adjusted for Pollen | **1.46** | **1.37** | **1.56** |  | **1.40** | **1.28** | **1.54** |
| + NOx | 1.09 | 1.00 | 1.19 |  | **1.27** | **1.10** | **1.47** |
| + O_3_ | **1.10** | **1.01** | **1.20** |  | **1.23** | **1.06** | **1.43** |
| + PM_2.5_ | **1.11** | **1.02** | **1.21** |  | **1.28** | **1.09** | **1.50** |
| + NOx, O_3_, PM_2.5_ | **1.21** | **1.11** | **1.31** |  | **1.28** | **1.12** | **1.46** |
| **Dyspnea** |  |  |  |  |  |  |  |
| Adjusted for Pollen | **1.20** | **1.12** | **1.28** |  | 1.09 | 0.96 | 1.23 |
| + NOx | 1.05 | 0.96 | 1.15 |  | 1.05 | 0.89 | 1.24 |
| + O_3_ | 1.04 | 0.95 | 1.14 |  | 1.03 | 0.87 | 1.20 |
| + PM_2.5_ | 1.02 | 0.93 | 1.11 |  | 0.98 | 0.83 | 1.16 |
| + NOx, O_3_, PM_2.5_ | 1.03 | 0.94 | 1.13 |  | 1.02 | 0.85 | 1.21 |
| **Dry cough** |  |  |  |  |  |  |  |
| Adjusted for Pollen | **1.14** | **1.06** | **1.22** |  | 0.97 | 0.87 | 1.08 |
| + NOx | **1.12** | **1.03** | **1.23** |  | 1.05 | 0.93 | 1.19 |
| + O_3_ | **1.10** | **1.01** | **1.20** |  | 1.06 | 0.93 | 1.20 |
| + PM_2.5_ | 1.08 | 0.98 | 1.18 |  | 1.06 | 0.88 | 1.29 |
| + NOx, O_3_, PM_2.5_ | **1.11** | **1.01** | **1.22** |  | 1.05 | 0.93 | 1.20 |
| **Increased use of allergy medication*** | | | | | |  |  |
| Adjusted for Pollen | **1.75** | **1.60** | **1.91** |  | **2.04** | **1.71** | **2.43** |
| + NOx | **1.22** | **1.10** | **1.36** |  | **1.23** | **1.02** | **1.48** |
| + O_3_ | **1.18** | **1.06** | **1.31** |  | **1.22** | **1.01** | **1.47** |
| + PM_2.5_ | **1.15** | **1.03** | **1.28** |  | 1.15 | 0.94 | 1.40 |
| + NOx, O_3_, PM_2.5_ | **1.18** | **1.06** | **1.32** |  | 1.15 | 0.94 | 1.40 |
| **Increased use of bronchodilating medication*** | | | | | |  |  |
| Adjusted for Pollen | **1.21** | **1.11** | **1.32** |  | **1.27** | **1.11** | **1.45** |
| + NOx | 0.97 | 0.89 | 1.06 |  | **1.27** | **1.06** | **1.52** |
| + O_3_ | 0.96 | 0.87 | 1.05 |  | **1.27** | **1.06** | **1.51** |
| + PM_2.5_ | 0.96 | 0.88 | 1.05 |  | **1.30** | **1.07** | **1.58** |
| + NOx, O_3_, PM_2.5_ | 0.97 | 0.85 | 1.11 |  | **1.31** | **1.08** | **1.60** |
| For eye irritation/rhinitis, dyspnea, dry cough, allergy medication, “Yes” vs “No”. *For asthma medication, reporting “more than yesterday” vs “No”, “Less”, or “Same”. Results reported per 100 grains/m^3^ for pollen | | | | | | | |

## Table S2b City specific estimates of diary-reported **Δ**PEF associated with pollen exposure at lag 0-2 in single-, pairwise-, and multi-exposure models (per 100 grains/m^3^). Significant results are indicated with bold font.

|  | **Gothenburg (n=22)** | | |  | **Umeå (n=15)** | | |
| --- | --- | --- | --- | --- | --- | --- | --- |
|  |  | **95% CI** | |  |  | **95% CI** | |
| **ΔPEF_ev_** | **β** | **Lower** | **Upper** |  | **β** | **Lower** | **Upper** |
| Adjusted for Pollen | -0.71 | -1.61 | 0.20 |  | **-1.95** | **-3.22** | **-0.67** |
| + NOx | -0.67 | -1.60 | 0.27 |  | **-1.98** | **-3.25** | **-0.71** |
| + O_3_ | -0.65 | -1.56 | 0.25 |  | **-1.95** | **-3.22** | **-0.67** |
| + PM_2.5_ | -0.79 | -1.71 | 0.13 |  | -1.25 | -2.63 | 0.12 |
| + NOx, O_3_, PM_2.5_ | -0.79 | -1.73 | 0.16 |  | -1.25 | -2.63 | 0.14 |
| **ΔPEF_mo_** |  |  |  |  |  |  |  |
| Adjusted for Pollen | -0.24 | -0.99 | 0.52 |  | **-2.04** | **-3.08** | **-1.00** |
| + NOx | -0.08 | -0.85 | 0.69 |  | **-2.10** | **-3.14** | **-1.06** |
| + O_3_ | -0.17 | -0.93 | 0.59 |  | **-2.04** | **-3.08** | **-1.00** |
| + PM_2.5_ | -0.34 | -1.10 | 0.42 |  | **-1.95** | **-2.99** | **-0.90** |
| + NOx, O_3_, PM_2.5_ | -0.21 | -0.98 | 0.57 |  | **-1.97** | **-3.03** | **-0.92** |
| Adjusted for relative humidity and temperature. Results reported per 100 grains/m^3^ for pollen. | | | | | | | |

## Table S3 Interaction effects of air pollution at low and high levels of birch pollen exposure on symptoms and asthma medication usage and peak expiratory flow (ΔPEF). Statistically significant results (*p*-values <0.05) are indicated in bold font and results with *p*-values above 0.05 or below 0.1, are indicated in italic font.

|  |  |  | **Coefficient of the interaction term** | | | | | | |  |
| --- | --- | --- | --- | --- | --- | --- | --- | --- | --- | --- |
|  |  |  | | |  | | **95% CI** |  | |  |
|  | **Observations** | **OR** | | **Lower** | | **Upper** | | | ***p* for interaction** | |
| **Eye irritation/rhinitis** |  |  | |  | |  | | |  | |
| Pollen x NO_x_ | 2010 | 1.40 | | 0.97 | | 2.03 | | | 0.07 | |
| Pollen x O_3_ | 2010 | **1.45** | | **1.14** | | **1.84** | | | **0.00** | |
| Pollen x PM_2.5_ | 1981 | **1.41** | | **1.04** | | **1.92** | | | **0.03** | |
| **Dyspnea** |  |  | |  | |  | | |  | |
| Pollen x NO_x_ | 1970 | 1.34 | | 0.88 | | 2.03 | | | 0.17 | |
| Pollen x O_3_ | 1970 | *1.33* | | *0.99* | | *1.78* | | | *0.06* | |
| Pollen x PM_2.5_ | 1941 | **1.56** | | **1.10** | | **2.21** | | | **0.01** | |
| **Dry cough** |  |  | |  | |  | | |  | |
| Pollen x NO_x_ | 1970 | 0.94 | | 0.62 | | 1.42 | | | 0.78 | |
| Pollen x O_3_ | 1970 | 0.94 | | 0.71 | | 1.24 | | | 0.65 | |
| Pollen x PM_2.5_ | 1941 | -0.03 | | 0.69 | | 1.36 | | | 0.85 | |
| **Allergy medication** |  |  | |  | |  | | |  | |
| Pollen x NO_x_ | 1973 | 0.94 | | 0.57 | | 1.56 | | | 0.81 | |
| Pollen x O_3_ | 1973 | 1.11 | | 0.80 | | 1.55 | | | 0.53 | |
| Pollen x PM_2.5_ | 1944 | **1.72** | | **1.13** | | **2.64** | | | **0.01** | |
| **Bronchodilating medication** | |  | |  | |  | | |  | |
| Pollen x NO_x_ | 1968 | 0.98 | | 0.55 | | 1.73 | | | 0.94 | |
| Pollen x O_3_ | 1968 | *1.44* | | *0.98* | | *2.12* | | | *0.07* | |
| Pollen x PM_2.5_ | 1939 | 0.90 | | 0.56 | | 1.46 | | | 0.68 | |
| **ΔPEF_mo_** | **Observations** | **β** | | **Lower** | | **Upper** | | | ***p* for interaction** | |
| Pollen x NO_x_ | 1907 | -0.75 | | -3.74 | | 2.24 | | | 0.62 | |
| Pollen x O_3_ | 1907 | -1.72 | | -4.06 | | 0.62 | | | 0.15 | |
| Pollen x PM_2.5_ | 1888 | -0.23 | | -2.92 | | 2.47 | | | 0.87 | |
| **ΔPEF_ev_** |  |  | |  | |  | | |  | |
| Pollen x NO_x_ | 1873 | -0.43 | | -4.23 | | 3.37 | | | 0.83 | |
| Pollen x O_3_ | 1873 | -0.59 | | -3.14 | | 1.95 | | | 0.65 | |
| Pollen x PM_2.5_ | 1845 | 1.35 | | -1.92 | | 4.61 | | | 0.42 | |
| All exposure at lag 02. The results are reported per pollutant IQR: NO_x_ 16.4 µg/m^3^; O_3_ 15.2 µg/m^3^. PM_2.5_ 4.67 µg/m^3^. The cutoff separating “low” and “high” values is 100 grains/ m^3^.  Results from mixed models adjusted for relative humidity and temperature, with identification number and study center as random effects. | | | | | | | | | |  |

## Table S4 Sensitivity analysis: ORs associated with pollen exposure at lag 0-2 in individuals with poor asthma control (ACQ score higher than 1.5), who use* inhaled corticosteroids (ICS users), nasal Steroid users (NS users), and those who participated in all three waves, from mixed models (adjusted for NO_x_, O_3_ and PM_2.5_, with identification number and city as random effect). Significant results are indicated with bold font.

|  | **Total data (n=37)** | | | | |  | **ACQ >1.5 (n=18)** | | | |  | **ICS users (n=25)** | | |  |  | **NS users**  **(n=9)** | | |  |  | **Participated in all three waves (n=27)** | | |
| --- | --- | --- | --- | --- | --- | --- | --- | --- | --- | --- | --- | --- | --- | --- | --- | --- | --- | --- | --- | --- | --- | --- | --- | --- |
|  |  | **95% CI** | | | |  |  | **95% CI** | | |  |  | **95% CI** | |  |  | | **95% CI** | |  |  |  | **95% CI** | |
|  | **OR** | **lower** | | **upper** | |  | **OR** | **lower** | **upper** | |  | **OR** | **lower** | **upper** |  | **OR** | | **lower** | **upper** |  |  | **OR** | **lower** | **upper** |
| **Eye irritation/rhinitis** | **1.22** | **1.14** | | **1.31** | |  | **1.29** | **1.12** | **1.48** | |  | **1.23** | **1.13** | **1.33** |  | **1.44** | | **1.19** | **1.75** |  |  | **1.21** | **1.12** | **1.30** |
| **Dyspnea** | 1.05 | 0.97 | | 1.14 | |  | 0.99 | 0.87 | 1.12 | |  | 0.99 | 0.90 | 1.09 |  | 1.05 | | 0.86 | 1.29 |  |  | **1.09** | **1.00** | **1.19** |
| **Dry cough** | **1.12** | **1.04** | | **1.22** | |  | 1.15 | 0.99 | 1.33 | |  | 1.07 | 0.97 | 1.17 |  | 1.03 | | 0.86 | 1.23 |  |  | **1.13** | **1.04** | **1.23** |
| **Allergy medication** | **1.18** | **1.07** | | **1.30** | |  | 0.94 | 0.79 | 1.12 | |  | **1.13** | **1.02** | **1.26** |  | **1.44** | | **1.14** | **1.80** |  |  | **1.19** | **1.07** | **1.32** |
| **Bronchodilating medication** | 1.05 | 0.95 | | 1.16 | |  | 0.91 | 0.75 | 1.10 | |  | 0.99 | 0.92 | 1.08 |  | 0.98 | | 0.81 | 1.19 |  |  | 1.07 | 0.97 | 1.19 |
|  |  | | **95% CI** | |  |  |  | **95% CI** | |  |  |  | **95% CI** | |  |  | | **95% CI** | |  |  |  | **95% CI** | |
|  | **β** | **lower** | | **upper** | |  | **β** | **lower** | **upper** | |  | **β** | **lower** | **upper** |  | **β** | | **lower** | **upper** |  |  | **β** | **lower** | **upper** |
| **ΔPEF_ev_** | **-1.72** | **-2.65** | | **-0.78** | |  | -1.62 | -3.28 | 0.04 | |  | -0.72 | -1.89 | 0.45 |  | -1.02 | | -2.49 | 0.44 |  |  | **-1.72** | **-2.74** | **-0.71** |
| **ΔPEF_mo_** | **-1.48** | **-2.30** | | **-0.66** | |  | -1.31 | -2.79 | 0.16 | |  | -0.75 | -1.79 | 0.29 |  | -1.02 | | -2.58 | 0.54 |  |  | **-1.58** | **-2.48** | **-0.68** |
| *Regular use in the last month | | | | | | | | | | |  |  |  |  |  |  | |  |  |  |  |  |  |  |

## Figure S1 Daily average concentration of NO_x_, O_3_, and PM_2.5_ (3-day moving average)


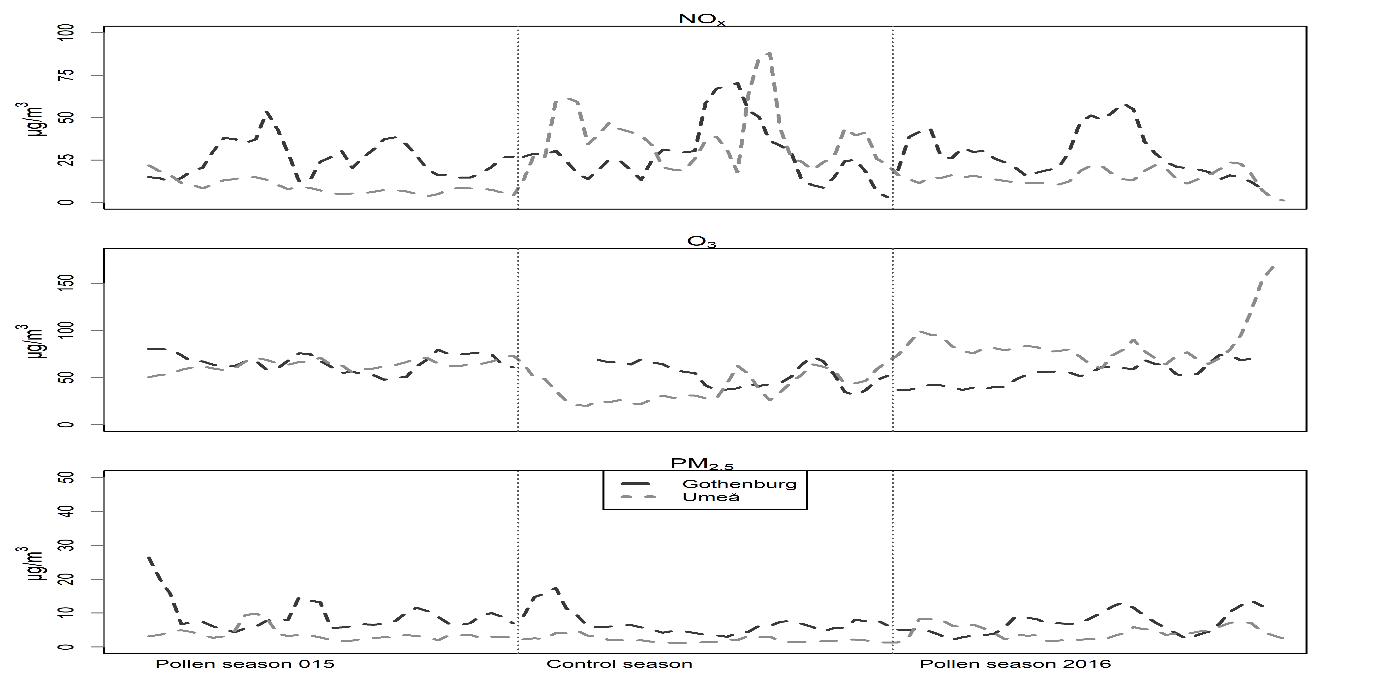


## Figure S2 Lag-association plots for the association between pollen, PEF and symptoms in unadjusted models, and models adjusted for PM, NO_x_ and O_3_

| 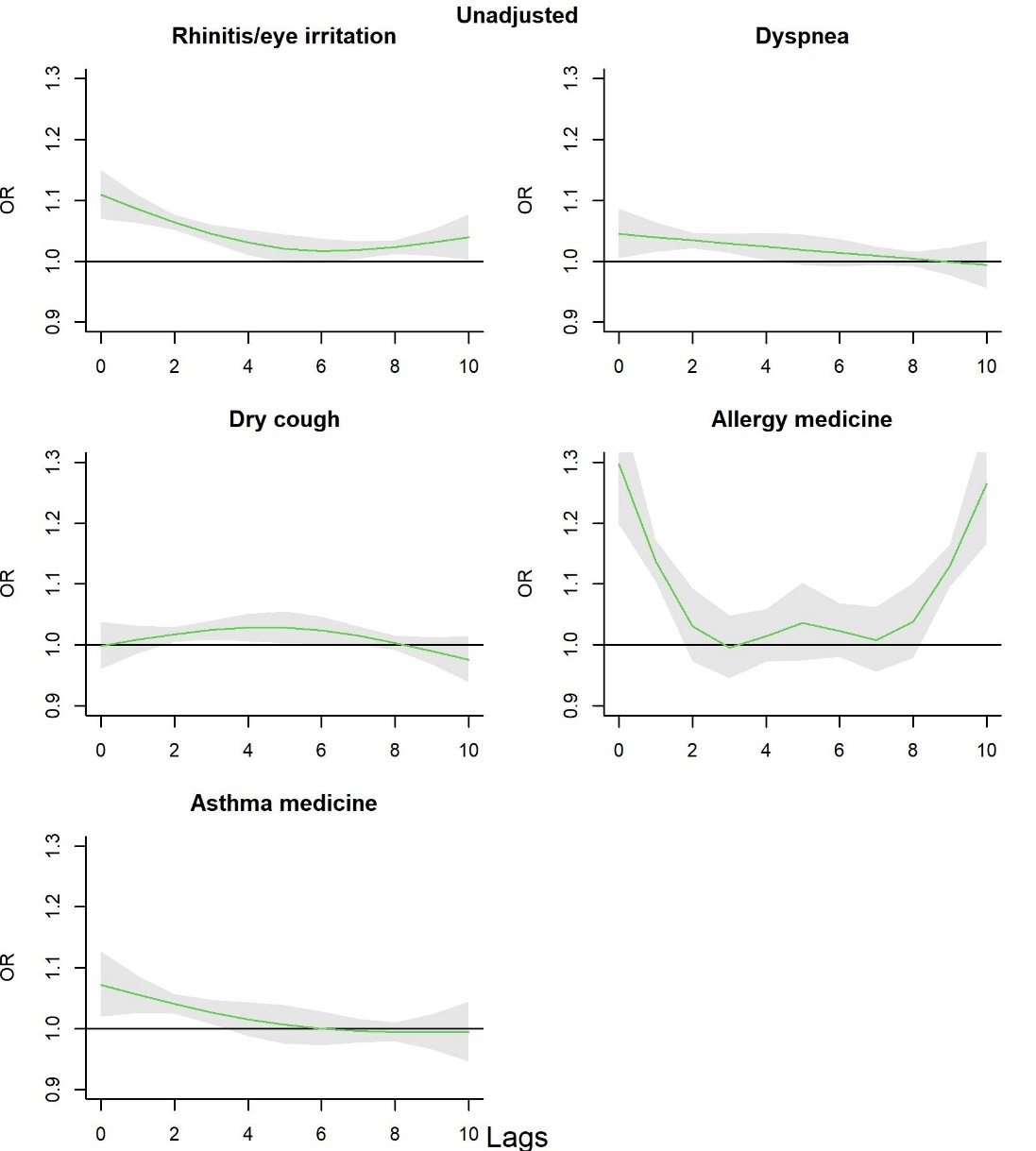 | 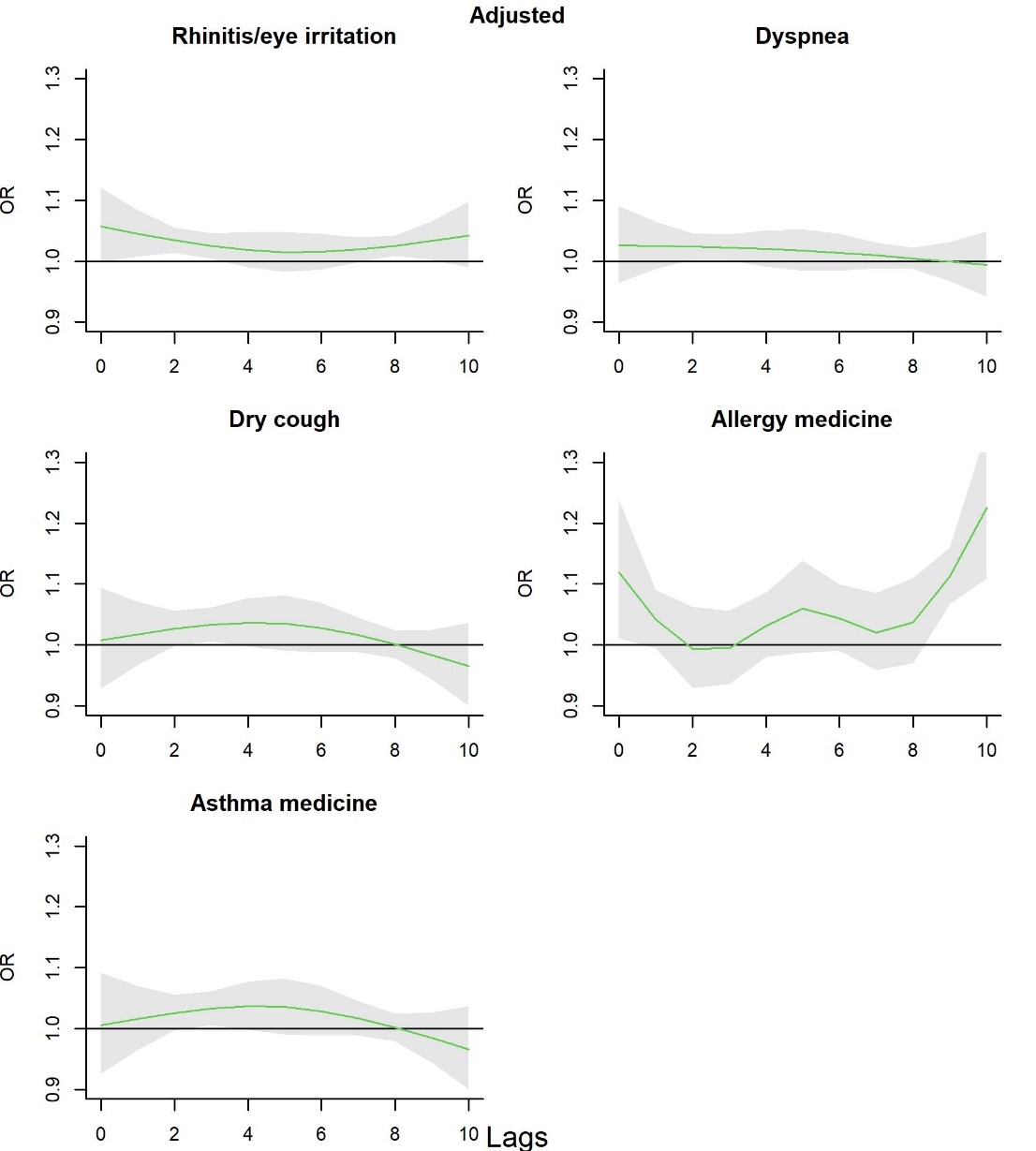 |
| --- | --- |
|  |  |
| 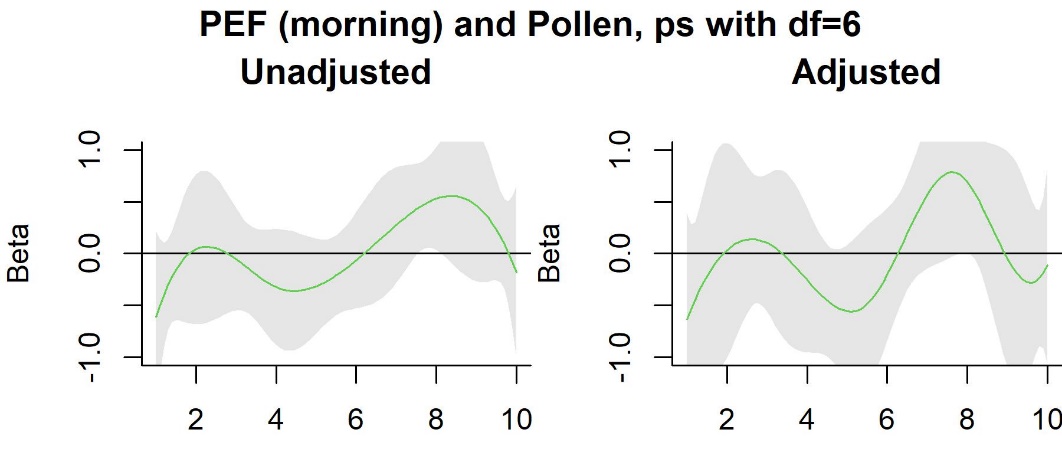 Lags Lags | 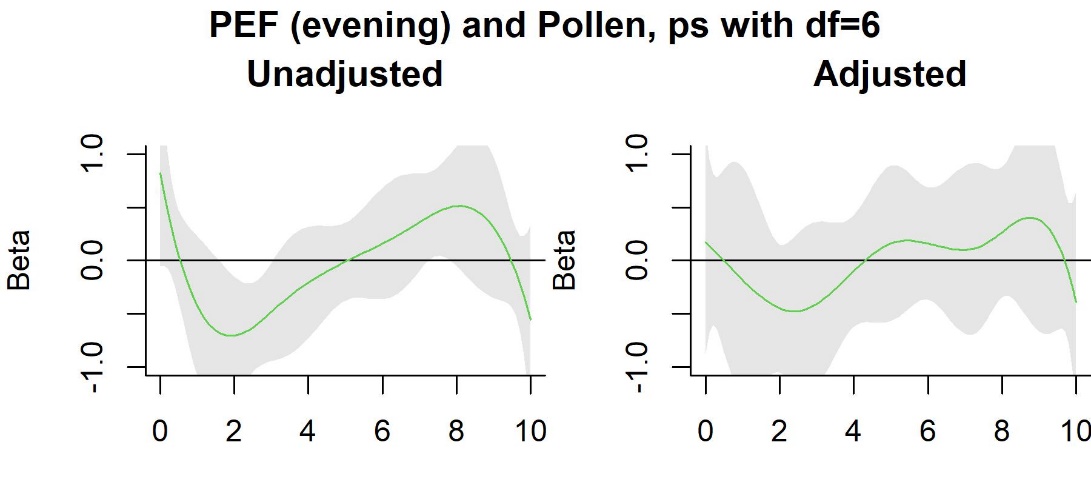  Lags Lags |

### DLNM Methodology

Observations from days where participants reported fever were excluded.

A stratum for each city and wave was constructed.

data$wave <- as.factor(data$wave)

data$City <- as.factor(data$City)

data$stratum <- as.factor(data$City:data$wave)

Crossbases with penalized spline (with 5-7 degrees of freedom) or knots at 1-3 places were created for the exposure variables

cb.pol <-crossbasis(data$l_Pollen, lag=c(1,10), argvar=list(fun="lin"), arglag=list(fun="ps", df=5), group=data$stratum)

cb.o3 <-crossbasis(data$O3, lag=c(1,10), argvar=list(fun="lin"), arglag=list(fun="ps", df=5), group=data$stratum)

cb.nox <-crossbasis(data$NOx, lag=c(1,10), argvar=list(fun="lin"), arglag=list(fun="ps", df=5), group=data$stratum)

cb.pm <-crossbasis(data$l_PM, lag=c(1,10), argvar=list(fun="lin"), arglag=list(fun="ps", df=5), group=data$stratum)

Models of symptom rates (binary outcomes) were tested at different adjustment levels

1. Pollen and gender

model1_2_1<- glmer(Dyspnea_f4 ~ cb1_2.pol+ sex + (1|ind), family=binomial(link="logit"), data=data)

1. Pollen, gender, temperature and relative humidity, time trend, Ozone, NOx, and PM_10_.

model1_2_3<- update (model1_2_1, .~. + temp02 + rh02 + pspline(time,df=3) + cb1_2.o3 + cb1_2.nox + cb1_2.pm )

And similarly for continuous outcomes

model1_2_1<- lmer(deltaPEF_k ~ cb1_2.pol + as.factor(gender) + (1|ind), data=data)

model1_2_3<- update (model1_2_1, .~. + temp02 + rh02+ pspline(time,df=3) + cb1_2.o3 + cb1_2.nox + cb1_2.pm )

The model results were illustrated using crosspred for pollen

pred1_2_1.pol <- crosspred(cb1_2.pol, model1_2_1, at=0:1, bylag=0.1, cumul=TRUE)

And for air pollutants

pred1_2_2.pol <- crosspred(cb1_2.pol, model1_2_3, at=0:1, bylag=0.1, cumul=TRUE)

### Crossbasis parameters for the bet-fitting models for each outcome

| **Outcome** | **Best unadjusted model**  **Best AIC^[[1]](#footnote-1)^ /R^2^ ^[[2]](#footnote-2)^** | **Best adjusted model**  **Best AIC /R^2^** | **Lag day (s) with significant effect of log(Pollen)**  **Unadjusted / adjusted** |
| --- | --- | --- | --- |
| Rhinitis/eye irritation | 1 knots/Ps^[[3]](#footnote-3)^, 7 df^[[4]](#endnote-1)^^[[5]](#footnote-4)^ | 1 knots/ Ps, 7 df | 0-4 /1-3 |
| Dyspnea | 1 knot / Ps, 7 df | 1 knot / Ps, 7 df | 0-4 / 2-3 |
| Dry cough | 1 knots/ Ps, 7 df | 1 knots/ 3 knots | 2-6 /3 |
| Allergy medication | 3 knots /3 knots | 3 knots / 3 knots | 0-1 /1 |
| Asthma medication | 1 knot / Ps, 7 df | 1 knot / Ps, 7 df | 1-3 /3 |
| PEF (morning) | -^[[6]](#footnote-5)^*/ Ps, 6 df | -*/ ps, 7 df | ns^[[7]](#footnote-6)^ / ns |
| PEF (evening) | -*/ ps, 7 df | -*/ ps, 6 df | 2-3 / ns |
| From GLMER and LMER mixed models with person as fixed effect and gender as random effects, adjusted models were also adjusted for climate and other pollutants. | | | |

## Figure S3 Predicted mean effects of pollen-air pollution interactions (in models with interaction p-values below 0.1, but over 0.05)
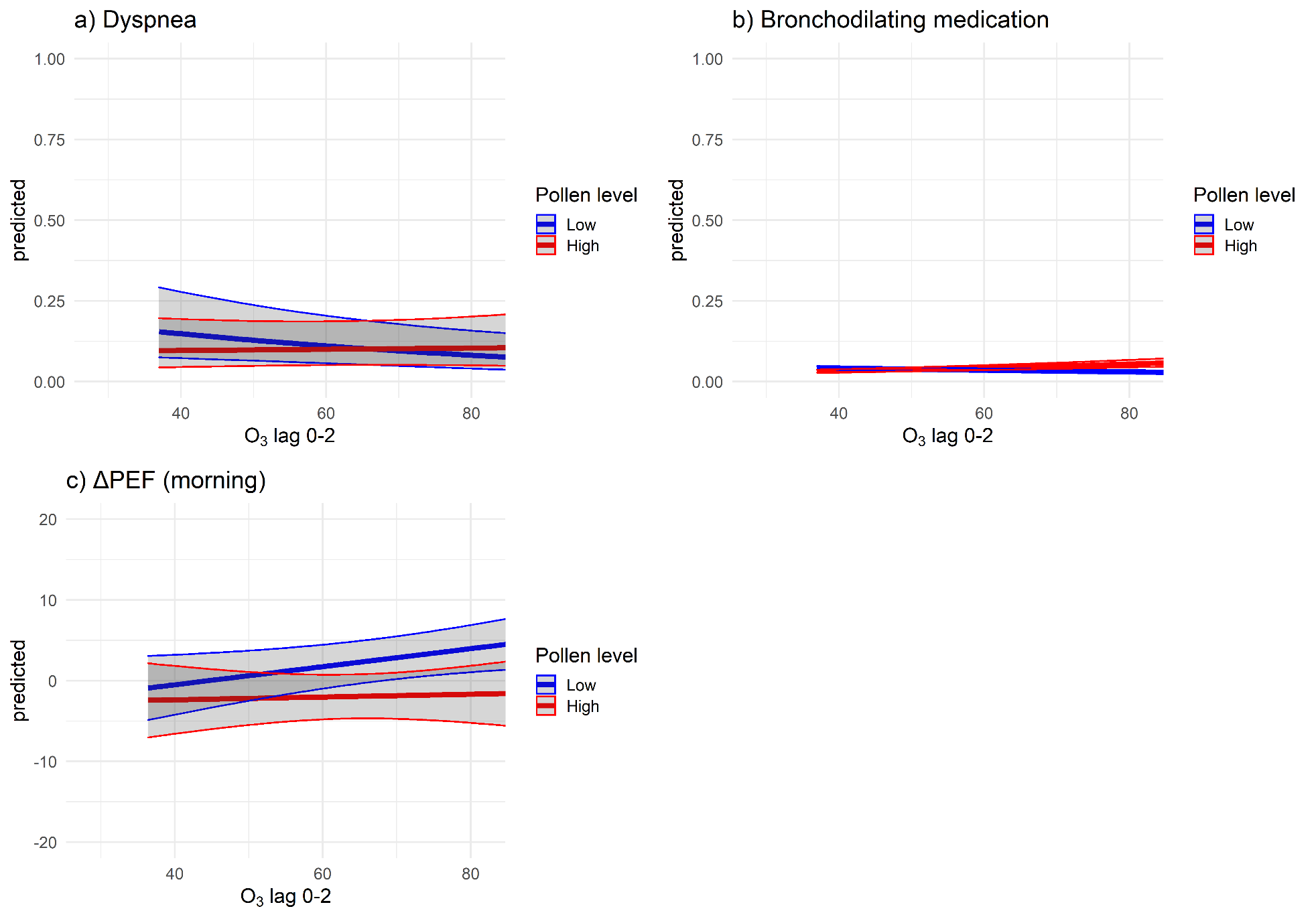


## Figure S4 Sensitivity analysis: Sex-stratified associations between for pollen and (a) symptoms and medication usage and (b) PEF (per 100 grains/m^3^) at lag 0-2 from mixed models (adjusted for NO_x_, O_3_ and PM_2.5_, with identification number and city as random effect)

| 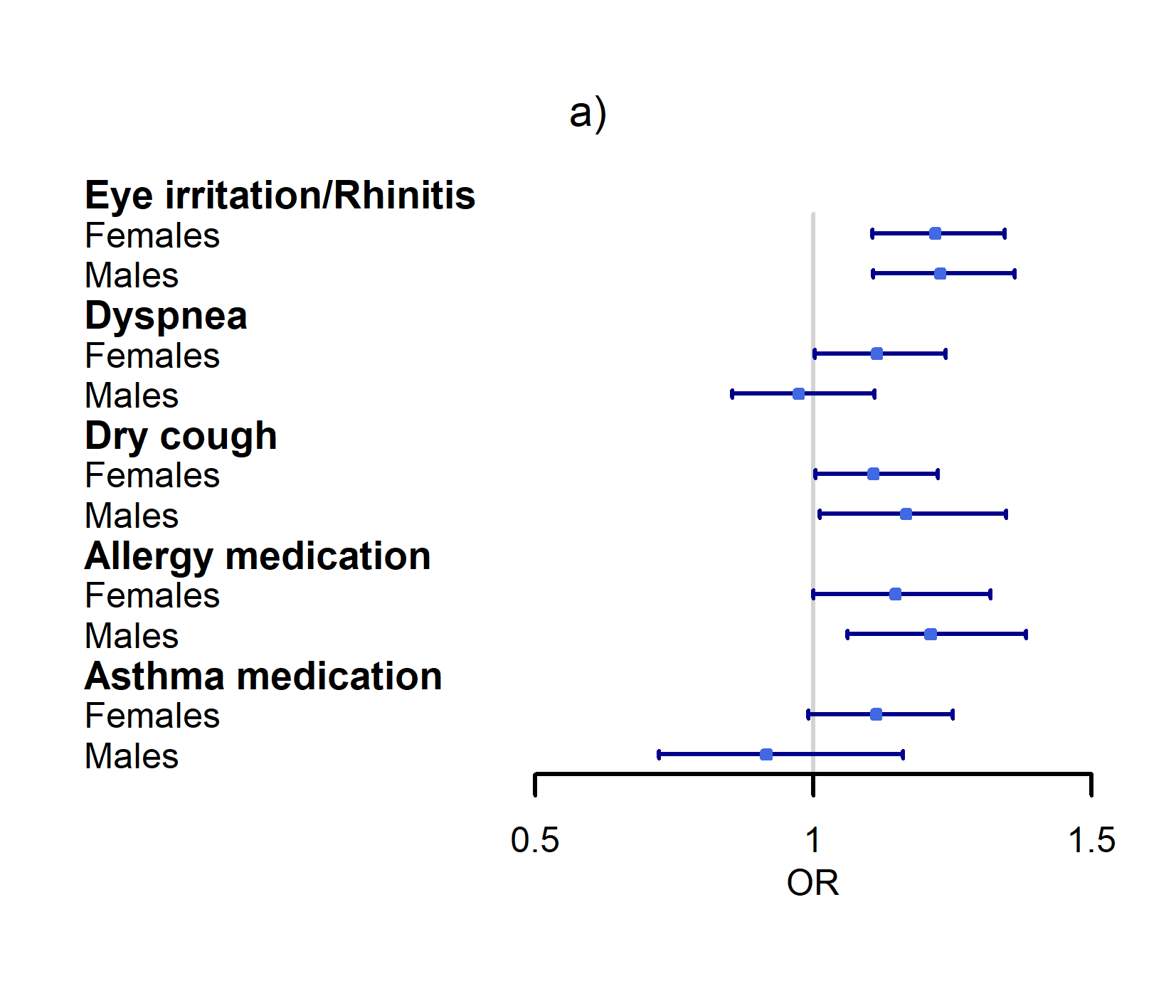 | 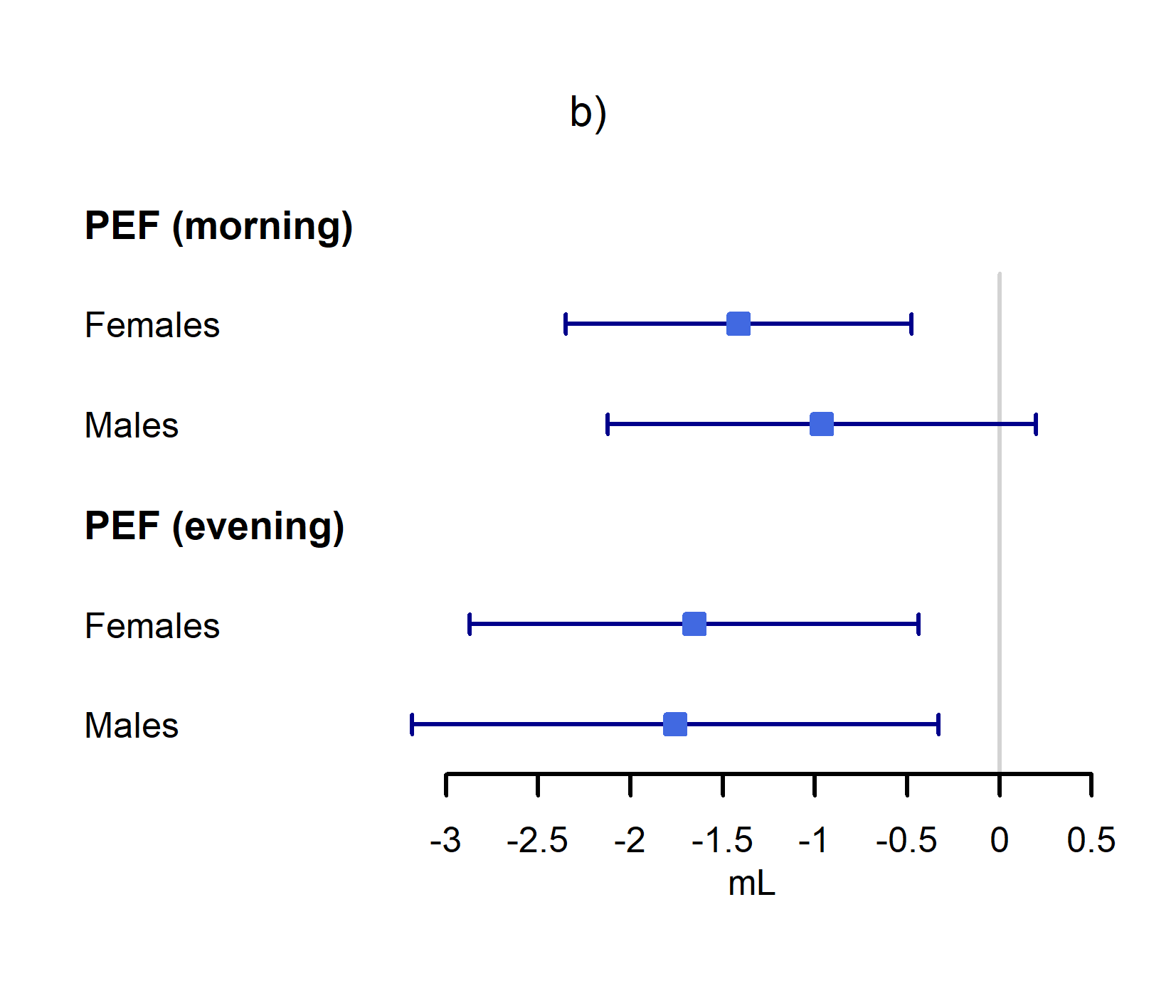 |
| --- | --- |

## Document D1 Symptom and peak expiratory flow (PEF) Diary example (English translation of the questions to the right).

|  | **Birch pollen study**  **Diary \| Asthmatic individuals**  PEF  -morning measure (#1/#2/#3)  Have you had a cold today? Yes / No  ________________________________________________________  Have you had a fever today? Yes / No  Have you had rhinitis or eye irritation today? Yes / No  Have you had wheeze or dyspnea today? Yes / No  ________________________________________________  Have you had a dry cough today? Yes / No  Have you taken medicine against your allergy today (Medicines, *NOT* natural remedies)? Yes / No  Have you inhaled bronchodilating medication today? (Compare with your normal dose) No / Less / Normal / More  Have you had less, same as normal, or more symptoms from your asthma today than yesterday? Less / Same / More  PEF  - evening measure (#1/#2/#3) |
| --- | --- |

1. Akaike´s information criterion [↑](#footnote-ref-1)
2. From conditional [↑](#footnote-ref-2)
3. Penalized spline [↑](#footnote-ref-3)
4. [↑](#endnote-ref-1)
5. Degrees of freedom [↑](#footnote-ref-4)
6. lmer does not provide AIC [↑](#footnote-ref-5)
7. Ns: No significant parameters [↑](#footnote-ref-6)
